# Differentiation of Human Mesenchymal Stem Cells into Insulin Producing Cells by Using A Lentiviral Vector Carrying *PDX1*

**DOI:** 10.22074/cellj.2016.3721

**Published:** 2015-07-11

**Authors:** Amir Allahverdi, Saied Abroun, Arefeh Jafarian, Masoud Soleimani, Mohammad Taghikhani, Fatemeh Eskandari

**Affiliations:** 1 Department of Hematology, Faculty of Medical Sciences, Tarbiat Modares University, Tehran, Iran; 2 Department of Clinical Biochemistry, Faculty of Medical Sciences, Tarbiat Modares University, Tehran, Iran

**Keywords:** *PDX1*, Diabetes Type I, Meneschymal Stem Cells

## Abstract

**Objective:**

Type I diabetes is an immunologically-mediated devastation of insulin producing cells (IPCs) in the pancreatic islet. Stem cells that produce β-cells are a new promising tool. Adult stem cells such as mesenchymal stem cells (MSCs) are self renewing multi potent cells showing capabilities to differentiate into ectodermal, mesodermal and endodermal tissues. Pancreatic and duodenal homeobox factor 1 (*PDX1*) is a master regulator gene required for embryonic development of the pancreas and is crucial for normal pancreatic islets activities in adults.

**Materials and Methods:**

We induced the over-expression of the *PDX1* gene in human bone marrow MSCs (BM-MSCs) by Lenti-*PDX1* in order to generate IPCs. Next, we examine the ability of the cells by measuring insulin/c-peptide production and *INSULIN* and *PDX1* gene expressions.

**Results:**

After transduction, MSCs changed their morphology at day 5 and gradually differentiated into IPCs. *INSULIN* and *PDX1* expressions were confirmed by real time polymerase chain reaction (RT-PCR) and immunostaining. IPC secreted insulin and C-peptide in the media that contained different glucose concentrations.

**Conclusion:**

MSCs differentiated into IPCs by genetic manipulation. Our result showed that lentiviral vectors could deliver *PDX1* gene to MSCs and induce pancreatic differentiation.

## Introduction

Diabetes afflicts more than 300 million people in the worldwide, accounting for approximately 12% of the world’s total health cost. Diabetes is classified two broad categories, type 1 (5-10%) and type 2 (90-95%) (1, 2). Type I diabetes is an immunologically-mediated devastation of the insulin producing cells (IPCs) in the pancreatic islet (3). Although insulin therapy is an ordinary treatment for type 1 diabetes, a regimen of several daily injections and need to inject for each meal may be difficult to achieve optimal glycemic control (4). Other treatments such as transplantation of the pancreas have disadvantages; such as the lack of donors for islet transplantation and immune rejection (5). Stem cells that produce β-cells are a new promising tool.

Embryonic stem cells (ESCs) are pluripotent cells that can differentiate into three germ layers. These cells have the capability to differentiate into insulin producing cells, however they have increased the raise risk for tumor formation in the recipient (6). Adult stem cells such as mesenchymal stem cells (MSCs) are multipotent cells with self-renewal capability to differentiate into ectodermal, mesodermal, and ectodermal tissues (7). Human MSCs are isolated from somatic tissues including bone marrow (BM), adipose tissue or fetal tissue, such as amniotic fluid (AF), Wharton’s jelly and umbilical cord blood (8).

*Pancreatic and duodenal homeobox factor 1 (PDX1)* is a transcriptional factor necessary for embryonic development of the pancreas. *PDX1* plays a distinct role in proliferation and differention. Heterozygote missense and frame shift mutations result in decreased secretion of insulin from β-cells which creates maturity onset diabetes of the young (MODY4) (9).

Lentiviral vectors result in high transmission efficiency, stable gene expression in target cells and are useful tool for genetic manipulation (10). In this study we have used a lentiviral vector to deliver the *PDX1* gene into MSCs to generate IPCs. After transduction we measured insulin and C-peptide production in these cells. *INSULIN* and *PDX1* gene expressions were evaluated by real-time polymerase chain reaction (RT-PCR) and immune cytochemical analyses.

## Materials and Methods

### Cell isolation and culture analysis of human mesenchymal stem cell

We obtained three healthy samples for BM transplantation from healthy donors (age 20- 40 years). These samples were obtained after patients were informed about the study and signed informed consents and permission from the local Ethics Committee at Taleghani Hospital (Tehran, Iran). BM samples (5 ml) were obtained from the posterior superior iliac crest of each patient and transferred to clean heparinized collection tubes. Samples were diluted with phosphate-buffered saline (PBS, Merck, Germany) at 1:1 ratio to which 3-4 ml Ficoll (Ficoll-paque, 1.073 g/ml, Pharmacia Uppsala, Sweden) was added. Samples were subsequent - ly centrifuged for 20 minutes at 400 g. The mononuclear cells located at the plasma: density gradient medium interface were collected and transferred to a new tube, PBS was added to the cells tubes were centrifuged for 5 minutes at 400 g, after which the supernatant was discarded. The cells were cultured in 5 ml of Dulbecco’s Modified Eagle’s Medium-low glucose (DMEM-LG, Gibco, USA) supplemented with 10 % fetal bovine serum (FBS, Gibco, USA), and 100 U/ml of penicillin/streptomycin (Gibco, USA), after which they were incubated at 37˚C in 5% humidified CO_2_. After 3-4 days, we replaced half of the medium with fresh medium to remove any non-adherent cells. When the cells reached 80-90% confluency, they were collected by treatment with 0.25% trypsin (Gibco, USA) and 1 mM ethylenediaminetetraacetic acid (EDTA) for 2-5 minutes at 37˚C. After centrifugation, MSCs were cultured in 75 cm^2^ flasks until confluent.

### Flow cytometry analysis of human mesenchymal stem cells

After the fourth passage, MSCs were detached by trypsinization. The cells were washed with PBS, and incubated with labeled phycoerythrin (PE)-conjugated monoclonal antibodies (Invitrogen, USA) at the dilutions recommended by the manufacturer at 4˚C for 25 minutes in the dark. The antibodies used were cluster of differentiation (CD) markers; CD90, CD34, CD105, and CD31-PE (BD Biosciences, USA). PE-labeled isotype-matched immunoglobulin was the negative control. The labeled cells were analyzed on a FACS Caliber flow cytometer (Becton-Dickinson, FACScan, San Jose, CA, USA).

### Adipocyte and osteoblast differentiation of mesenchymal stem cells

MSCs at passage 4 were used for osteoblast and adipocyte differentiation. For adipocytes, cells were placed in medium containing LDMEM (Bioidea, Iran), 10% FBS, 106 M dexamethasone (Sigma, USA), and 0.05 mg/dl ascorbic acid (Merck, Germany). After two weeks, differentiated adipocyte cells were stained with oil red O (Sigma, USA).

Cells were differentiated into osteoblasts in medium that contained L-DMEM, 10% FBS, 10^-8^ M dexamethasone, 10 mM β-glycerophosphate (Merck, Germany), and 0.05 mg/dl ascorbic acid. After 3 weeks, differentiated osteoblasts cells were stained with alizarin red (Sigma, USA).

### Oil-red O staining

Cells were washed with PBS and fixed with 4% para-formaldehyde (Merck, Germany) for 20 minutes, after which 60% isopropanol (Merck, Germany) was added. After 5 minutes, the isopropanol was removed. Oil red O was dissolved in 99% isopropanol and added to cells. The cells were allowed to incubate for 15 minutes at room temperature. The dye was subsequently removed and cells were washed with PBS until the water rinsed off clear.

### Alizarin-red staining

The cells were washed with PBS, fixated with 4% para-formaldehyde, immersed in a 1% alizarin red solution for 10 minutes and rinsed with distilled water (DW).

### Plasmids

EX-M0942-Lv105 plasmid that carried the *PDX1* gene was purchased from (Genecopoeia, USA). PsPAX2 and pMD2.G vectors for viral packaging were purchased from (Invitrogen, USA). These vectors were transformed in DH5α. Plasmids were purified with a kit (real-biotech, plasmid mini kit, Taiwan). One single copy of EX-M0942-Lv105 contained 7730 bp. We used a software program available at: http://cels.uri.edu/gsc/cndna.html to calculate the copy number of the plasmid *PDX1*. There were 7.23×10^10^ copies per μ liter. This calculation was needed for viral particle titration. Serial dilutions of the plasmid (7.23×10^7^-7.23×10^9^) were prepared, after which we created a *PDX1* plasmid standard curve for determine the copy number of integrated lentiviruses.

### Production of lentiviral vector in HEK-293 cells

HEK-293T cells that had density of 4×10^6^/ml were seeded in 100 mm dishes (JET Biofil, China) that contained L-DMEM supplemented with 10% FBS. When the cells reached 80% confluency, transfection with *PDX1* plasmid and the two packaging viral vectors (pMD2.G and psPAX2) was performed according to the calcium-phosphate protocol. The cells supernatants were collected every 24 hours and fresh medium that contained serum was added to the cells. After 72 hours, the total viral medium was centrifuged for 10 minutes at 2100 g and filtered through a 0.45 micron syringe filter. Virus’s particles were concentrated by poly ethylene glycol (PEG, Sigma, USA) 50% and Nacl 5 M (Merck, Germany). The supernatant was incubated for 16-20 hours on a shaker at 4˚C. After which the samples were centrifuged for 10 minutes at 4100 g and a temperature of 4˚C. Sediment dissolved in centrifuged in 1 ml of DMEM-F12 medium and aliquted into sterile 1.5 μl microtubes. Cells were collected and *PDX1* gene expression examined by PCR and Western blot to confirm virus production.

### Titration lenti-vector by real-time polymerase chain reaction

HEK-293T cells were seeded at density of 6×10^4^ /mL per well in 12-well tissue culture plates. We added 50 and 100 μl of concentrated virus to the cells. After 16 hours, the culture medium was replaced with fresh DMEM media supplemented with 10% FBS. After 96 hours, we extracted the DNA from traduced cells with a Spin Blood Mini Kit (Invisorb®, Germany). Extracted DNA was stored at -20˚C until use. In quantitative real-time PCR (qPCR), a standard curve was generated by using 10 log that included a serial dilution (7.23×10^7^-7.23×10^9^ particles/μl) of plasmid EX-M0942-Lv105 which contained the puromycin resistance gene sequence. We used the Applied Biosystems Step One software v2.2 to calculate the standard curve. Recombinant DNA (unknown sample) extracted from HEK-293 and standard samples were run on Applied Biosystems Step One (Applied Biosystems, USA) with the SYBR-Green kit (Takara, Japan). Specificities of the products were evaluated by melting curve analysis. We used primer specific for the puromycin resistance gene to quantify the copy numbers of viral particles (Table 1). qPCR values are mean ± standard error of mean (SEM).

**Table 1:** Primer sequences and conditions for real time-polymerase chain reaction


Target genes size (bp)	Primers (forward, reverse)	Annealing TM (˚C)

PDX1	F: 5΄ATGGATGAAGTCTACCAAAGC 3΄	60
159	R: 5΄CGTGAGATGTACTTGTTGAATAG 3΄	
INSULIN	F: 5΄GAACGAGGCTTCTTCTACAC 3΄	59
143	R: 5΄ACAATGCCACGCTTCTG 3΄	
GAPDH	F: 5΄GACAAGCTTCCCGTTCTCAG 3΄	58
166	R: 5΄GAGTCAACGGATTTGGTCGT 3΄	
PRUMYCIN	F: 5΄TAAATATAGTCAATGTCCCTCAGC 3΄	58
174	R: 5΄TGTGGTTCTGTGTTGGTAGC 3΄	


### Transduction of mesenchymal stem cells

We seeded 5×10^5^ /well passage-4 human MSCs that were 70-80% confluent in a six-well plate. MSCs were transduced with medium that contained viral particles at 37˚C for 6-8 hours by the multiplicity of infection (MOI) of 50 and then replaced with fresh L-DMEM that contained 10% FBS. Medium replacement was performed two days per weeks.

### Reverse transcription polymerase chain reaction and real-time polymerase chian reaction

Total RNA was extracted from transfected HEK- 293 cells by Trizol (Invitrogen, USA) according to the manufacturer’s instructions. Total RNA was extracted at days 7, 14 and 21 post infection from MSCs. cDNA was synthesized from the RNA template according to the instructions below. First strand cDNA was synthesized from the RNA template. A total of 50 ng RNA was reverse transcribed by random hexamer primer using high capacity cDNA Synthesis Kit (Intron, Korea) at 42˚C for 60 minutes and 70˚C for 5 minutes. PCR reaction was performed in 25 μl final volume that contained 20.75 μl PCR master mix (Vivantis, Malaysia), 0.25 μl Taq polymerase (Vivantis, Malaysia), 2 μl *PDX1* forward and reverse primers (Table 1) and 2 μl cDNA. Amplification conditions were initial denaturation at 95˚C for 3 minutes and followed by 35 cycles at 60˚C and extension at 72˚C for 1 minute. Real-time PCR was performed using the Applied Biosystems Step One (Applied Biosystems, USA) with a SybrGreen Master Mix Kit (Takara, Japan) and performed in 10 μl volume that contained 1 μl cDNA and 1 μl primers (Table 1). *GAPDH* was used as a reference gene. The device Program was primary denaturation at 95˚C and annealing at 60˚C for 40 cycles. The qRT-PCR values are expressed as mean ± SEM of three independent real-time PCR experiments.

### Western blot analysis

We used Western blot analysis to measure of *PDX1* expression in transfected HEK-293. Total protein was extracted from transfected and non-transfected HEK- 293 by radioimmuno precipitation assay (RIPA) buffer that contained 10 mM tris-HCl (pH=8), 1% nonyl phenoxypolyethoxylethanol (NP-40), 10% glycerol, 0.1% sodium dodecyl sulfate (SDS), 1 mM EDTA and 100 mM Nacl with protease inhibitor cocktail (Roche Diagnostic, GmbH, Germany). There were 50 μg of lysates loaded into each well of a 15% SDSpolyacrylamide (SDS-PAGE, Merck, Germany) gel for electrophoresis analysis. After transfer of the separated proteins onto nitrocellulose membrane (Amersham Biosciences, USA) the membrane was blocked with 3% nonfat skim milk in a tris-buffered solution with 0.1% Tween 20 (TBST, Sigma, USA) for 2 hours, after which it was incubated overnight at 4˚C with mouse anti-human *PDX1* primary antibody (#ab84987, Abcam, Cambridge, MA, USA). The membrane was washed three times with TBST, then incubated with polyclonal anti-mouse horseradish proxidase-conjugated (HRP) secondary antibody (1:500, #AP8036, Razi Biotech, Iran) for 1hour at room temperature. After washing three times with TBST, the bands were visualized using enhanced chemiluminescence (ECL) reagent (Ariyatous Biotech, Iran) according to the manufacturer’s instructions.

### 3-(4, 5-dimethylthiazol-2-yl)-2,5-diphenyltetrazolium bromide (MTT) assay

We performed the MTT assay to assess viability of MSCs during differentiation to IPCs. 105 transduced cells per well on days 0, 7, 14 and 21were seeded into 96-well. The cells were incubated for 24 hours at 37˚C. The supernatant was removed and freshly prepared MTT (5 mg/ml) solution was added to the cells. The cells were subsequently incubated 4 hours at 37˚C. After the incubation period, 100 μl dimethyl sulfoxide (DMSO) was added to the wells and were incubated for 20 minutes at room temperature. The optical density (OD) was read at a 570 nm wavelength. Viability compared to control cells was calculated.

### Immunostaining

For immunocytochemical analysis, differentiated cells were scraped at day 21 and cells were washed twice in cold 2% FBS-PBS, then diluted in 100 ml of cold 1% bovine serum albumin in phosphate-buffered saline (BSA-PBS). 105 cells were fixed on slides by cytocentrifugation (Shandon Cytospin, USA). Cells were coated on the slides and fixed with 4% paraformaldehyde (PFA, Merck, Germany) for 20 minutes at room temprature. Then, cells were permeabilized with 0.2% Triton (Merck, Germany) 100-X for 5 minutes. Slides were washed twice and incubated in 5% goat serum for 45 minutes. The primary antibody were mouse anti-human insulin (1:100# ab7760, Abcam, Cambridge, MA, USA), mouse anti-human Neurogenin3 (1:100# ab87108 Abcam, Cambridge, MA, USA) and mouse anti-human *PDX1* (1:200#ab84987 Abcam, Cambridge, MA, USA). Cells were washed 3 times with PBS with Tween 20 (PBST) and incubated for 45 minutes at room temperature with fluorescence-labeled secondary antibody, including fluorescein isothiocyanate (FITC)-labeled goat anti-mouse IgG (1:50, #AF8032, Razi Biotech, Iran) and Texas red-labeled goat anti-mouse IgG (1:100, #ab5884). Nuclear DNA was stained with 0.1 μg/ml of blue-fluorescent 4΄, 6-Diamidino-2-phenylindole (DAPI, Sigma, USA) at 30˚C for 5 minutes. The slides were visualized under a fluorescence microscope (Nikon, USA).

### Insulin and C-peptide secretion and content

At 14 days after induction, the cells were washed with PBS and incubated in Krebs Ringer bicarbonate (KRB) buffer without glucose for 2 hours. Then, the cells were incubated in KRB that contained 5.5, 15 and 25 mM glucose at 37˚C. After incubation, cells were centrifuged and the supernatant collected and stored at -70˚C until the assayed. In order to measure intracellular insulin and C-peptide, total protein was extracted from the cells by lysis buffer and protease inhibitor, then sonicated three times for 15 seconds each at 40 W and centrifuged at 12000 rpm for 15 minutes at 4˚C. The supernatant was collected and kept at -70˚C utile use. Insulin and C-peptide were determined by enzyme immunoassay ELISA kit (#10-1132-01, #10-1141-01, Mercodia, Uppsala, Sweden). The conversion factors for insulin were 1 μg/ml=23 mU/l, 1 mU/l= 6.0 pmol/l and for C-peptide, 1 μg/l corresponded to 331 pmol/l.

### Statistical analysis

The data were analyzed by Graph Pad Prism 5 (GraphPad, La Jolla, CA, USA). One-way ANOVA followed by Bonferroni’s post-hoc test for comparison of experimental groups with the control was used to compare results. P values<0.05 were considered statistically significant.

## Results

### Cell culture and characterization of human bone marrow mesenchymal stem cells

**Fig.1:**
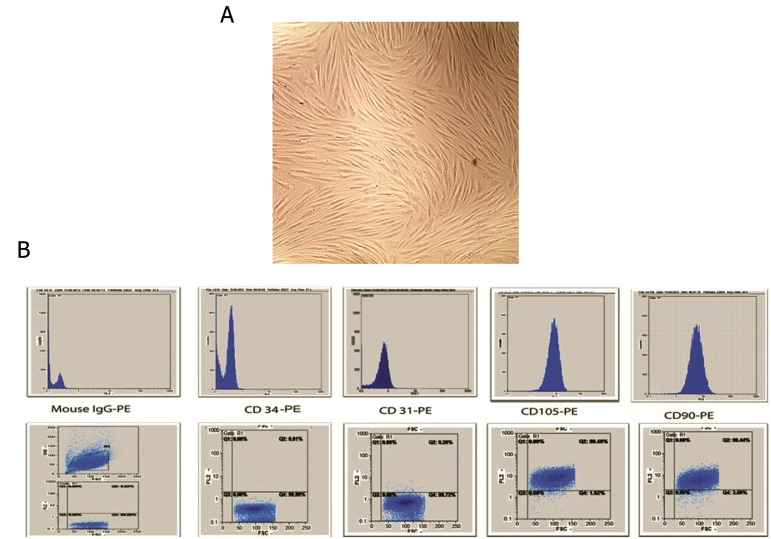
Morphological appearance of cultured cells and Flow cytometric analysis. A. Mesenchymal stem cells (MSCs) showed a spindleshaped fibroblastic morphology and B. Flow cytometry analysis of the MSC surface markers. These cells were positive for CD105 and CD90 and negative for CD31 and CD34. CD; Cluster of differentiation and PE; Phycoerythrin.

**Fig.2:**
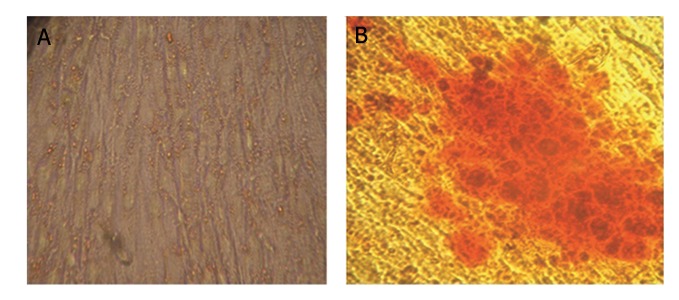
Multilineage differentiated of mesenchymal stem cells (MSCs). MSCs were differentiated toward adipogenic and osteogenic lineages by induction media. A. After two weeks adipogenic differentiation was assessed by oil red O staining and B. Osteogenic differentiation was analyzed after three weeks by alizarin red staining.

We collected BM samples from healthy donors. Mononuclear cells were obtained through density centrifugation by using a ficoll-paque solution. Non-adherent cells were removed at 3-4 days after primary culture and adherent cells were reached at 90% confluency at day 21. Then, the cells were seeded into a plastic flask. Primary MSCs showed a characteristic spindle-shaped morphology (Fig.1A). Flow cytometry analysis showed that sub cultured MSCs expressed high levels of CD105 and CD90 and low levels of CD31 and CD34 (Fig.1B). MSCs were successfully differentiated into osteogenic and adipogenic lineages. We observed osteogenic differentiation after 21 days of culture in osteogenic media as confirmed by alizarin-red staining. Adipocytes cells were confirmed by oil-red O staining at day 14 (Fig.2).

### Production of LV- *PDX1* in HEK 293 cells

**Fig.3:**
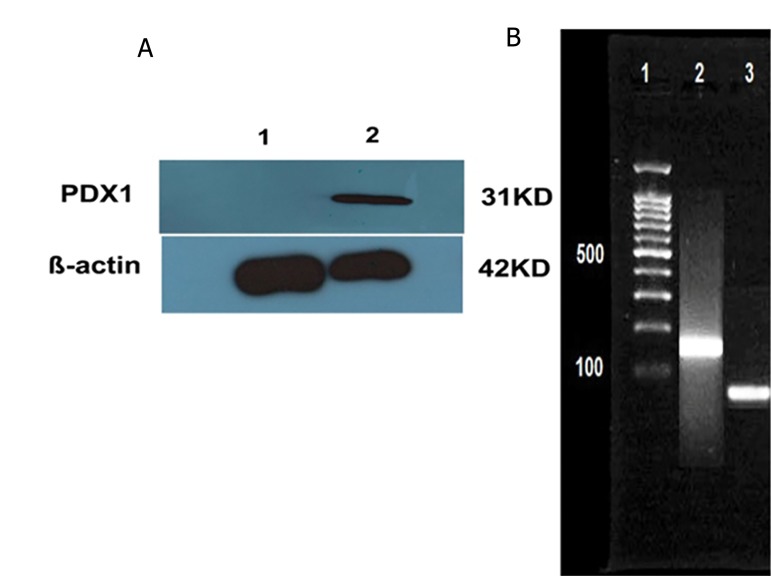
Confirmation of lentiviral transfection. A. Western blotting was used to detect *PDX1* protein (31 kD) and β-actin (42 kD) and B. Polymerase chain reaction analysis of *PDX1* gene. Lane 1; DNA ladder (100 bp), Lane 2; Transfected HEK 293 cells and Lane 3; Untransfected HEK-293.

The LV-*PDX1* vector was amplified and packaged in HEK-293 cells. Virus production was confirmed by detection of *PDX1* in HEK-293. RNA was extracted from transfected cells and cDNA synthesized. PCR analysis was performed to examine expression *PDX1* in the transfected cells (Fig.3A). We examined expression of the *PDX1* protein by Western blot in transfected cells (Fig.3B).

### Titration of lentivector by quantitive polymerase chain reaction (qPCR)

**Fig.4:**
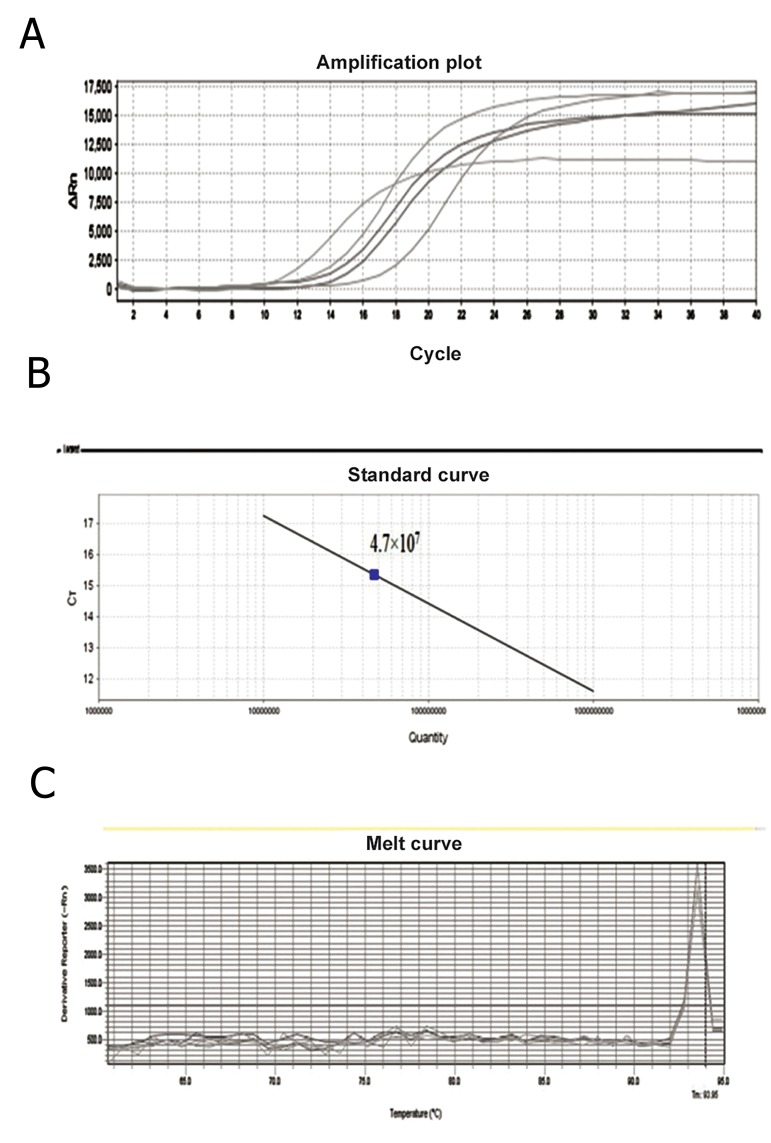
Quantitation of lentivrus vector samples by real-time polymerase chain reaction. A. Amplification plot of samples with each dilution represented in order from left to right on the graph, B. Standard curve for quantification of viral samples. Three 10-fold-dilutions (7.23×10^7^-7.23×10^9^ copies of the plasmid/μl) were used. The viral titer was 4.7×10^7^ particles/μl and C. Melting curve analysis of the samples. The products from the vector and viral samples had the same melting point peak at 93.85˚C.

To perform titration by quantitative real-time PCR, plasmid EX-M0942-Lv105 that contained the puromycin resistance gene and puromycin primers was used to derive the standard curves. The number of lentiviral vector copies was measured by the standard curve was created automatically with Applied Biosystems Step One v2.0 software in each run by plotting the Ct number against the copy numbers of each standard and quantification of viral DNA for unknown samples was inferred from the regresTo perform titration by quantitative real-time PCR, plasmid EX-M0942-Lv105 that contained the puromycin resistance gene and puromycin primers was used to derive the standard curves. The number of lentiviral vector copies was measured by the standard curve was created automatically with Applied Biosystems Step One v2.0 software in each run by plotting the Ct number against the copy numbers of each standard and quantification of viral DNA for unknown samples was inferred from the regression line (Fig.4A, B). Melting curve was generated to determine melting point of the products. Melting point was 93.85˚C. We observed that no contaminating products were present in this reaction (Fig.4C).

### Morphological changes of transduced mesenchymal stem cells

**Fig.5:**
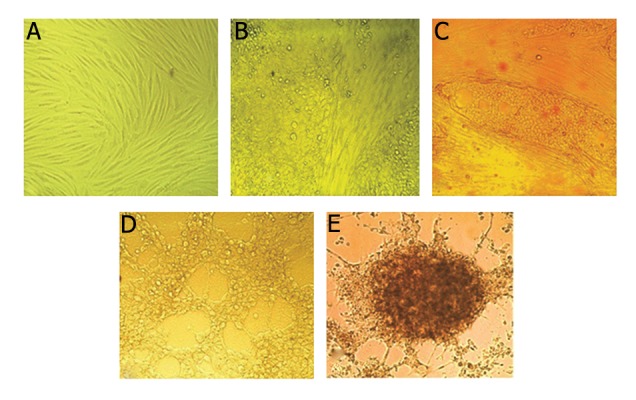
Morphological changes in transduced mesenchymal stem cells. A. The morphological change of transduced cells at day 1 (magnification ×200), B. Transduced cells at day 5, cells lost their spindle shape and became an endodermal cuboidal shape (magnification ×200), C. Transduct cells at day 7, Cells became round (magnification ×200), D. After 14 days cells finished their spherical shape and clumped together (magnification ×200) and E. The cells after 21 days differentiated into an islet-like cluster (magnification ×200).

We induced MSCs at passage 4 into islet-like cells in 21 days incubation. The MSCs exhibited a spindle-shape morphology before differentiation (Fig.5A), initial morphological changes were observed in the transduced cells at day 5 (Fig.5B). At day 7, spindle-shaped cells gradually clumped together (Fig.5C). At day 14, cells became aggregates and new islet-like clusters began to appear (Fig.5D). In addition, cell proliferation became slower and islet-like cluster formed on day 21 (Fig.5E).

### Pancreas-specific genes expressions

**Fig.6:**
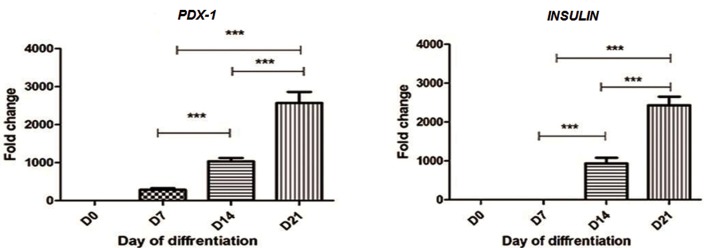
INSLIN and *PDX1* gene expressions at days 7, 14 and 21. RT-PCR analysis of *INSULIN* and *PDX1* genes. At three stage of differentiation (days 7, 14 and 21) into islet-like clusters in compared with undifferentiated MSCs (D0). The expression levels were normalized with GAPDH. Data are presented as mean ± SEM. Data were derived from three separate experiments and analysis after one-way ANOVA showed no significant changes. **; P<0.01, ***; P<0.0001, RT-PCR; Real time polymerase chain reaction, MSCs; Mesenchymal stem cells and SEM; Standard error of mean.

We analyzed the *PDX1* gene expression as a key pancreatic development transcription factor and *INSULIN* gene expression as an endocrine related marker by RT-PCR analysis. Analyses were performed in triplicate on cDNA samples of transduced cells at days 0,7,14 and 21. Our results showed that *PDX1* gene expressions increased at day 7^th^ with a gradual increase at days 14^th^ and 21^th^. (Fig.6, left). As illustrated in figure 6 right the differentiated MSCs at 14 days after induction expressed characteristic *INSULIN* gene expression which strongly increased at day 21. *INSULIN* gene expression was detectable at days 14 and 21 of differentiation. Our results showed that *PDX1* gene expression was determined earlier.

### Viability of mesenchymal stem cells during differentiation into insulin producing cells

**Fig.7:**
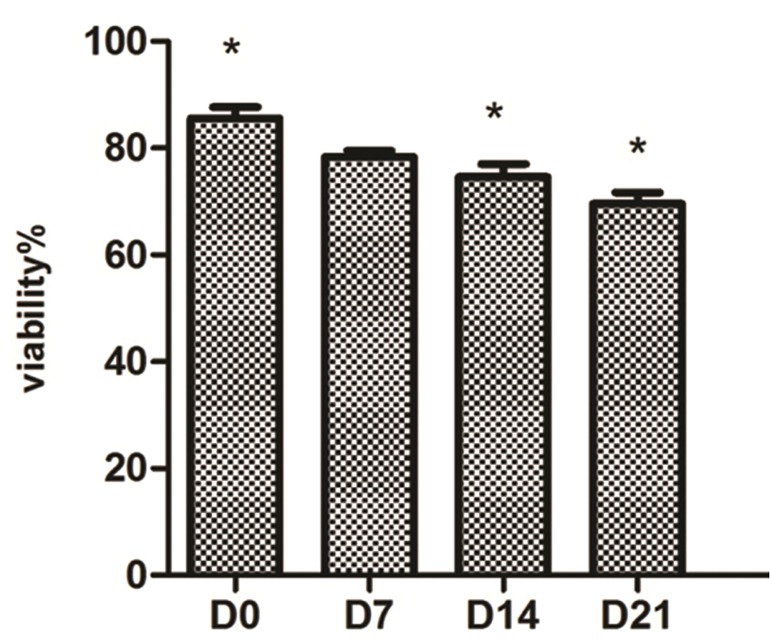
Relative cell viability of transduced mesenchymal stem cells (MSCs) on days 0, 7, 14 and 21. Cell viability was determined by 3-(4,5-dimethylthiazol-2-yl)-2,5-diphenyltetrazolium bromide (MTT) analysis. There was no difference between viability in cells on days 0 and 7, however viability of the cells at days 14 and 21 were reduced, which was statically significant. *; P<0.05. Data are presented as mean ± standard error of mean (SEM).

The MTT assay was performed on transduced cells at days 0, 7, 14 and 21 post-induction to determine the percentage of cell death during differentiation. Data were from three independent experiments. There was a statistically significant slight reduction (P<0.05) in IPC viability at days 7 and 21 of differentiation compared with undifferentiated day 0 MSCs (Fig.7).

### Immunofluorescence analysis

**Fig.8:**
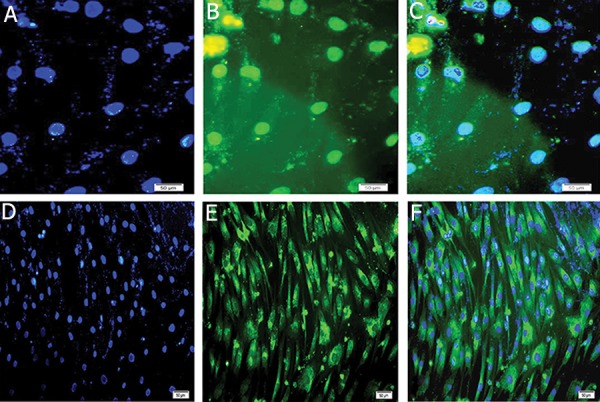
A, D. Counter staining of nucleus (blue) was performed by DAPI. Expressions of of *INSULIN* and *PDX1* in insulin producing cells (IPCs), B. FITC-conjugated *PDX1* antibody detected nuclei localization of *PDX1*, C. Merged image of nuclei immunostaining. E. FITC-conjugated insulin antibody detected cytoplasmic localization of insulin and F. Merged (magnification ×200).

*PDX1* and insulin protein were shown in transduced of MSCs *PDX1*^+^ using anti-*PDX1* and anti-insulin antibodies, which were represented as green dots in the immunofluorescence assay at day 21. Immunofluorescence analysis detected nuclei localization of *PDX1* and cytoplasmic localization of insulin. Counter-staining of the nucleus (blue) was performed by DAPI (Fig.8).

### Glucose-induced insulin and C-peptide secretion and content

**Fig.9:**
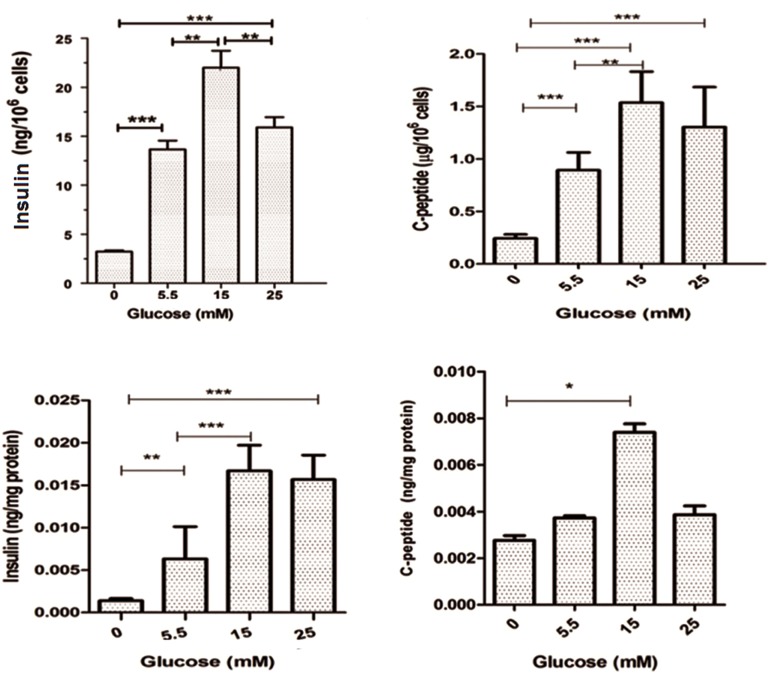
Insulin and c-peptide assay. A. Insulin secretion, B. C-peptide secretion in response to 0, 5, 15 and 25 mM glucose, C. Intracellular insulin content at each concentration of glucose versus of total cellular protein and D. Intracellular C-peptide content at each concentration of glucose versus of total cellular protein. Maximum amount of insulin and C-peptide were observed at 15 mM of glucose. Results are mean ± standard error of mean (SEM). **; P<0.01 and ***; P<0.001.

We examined IPCs in response to various glucose concentrations. The insulin level was 3.75 ± 0.70 ng/10^6^ cells prior to induction. At a concentration of 5.5 mM glucose, the insulin level was 14.32 ± 1.25 ng/10^6^ cells; at 15 mM, it was 22.35 ± 2.3 ng/10^6^ cells; and at 25 mM of glucose, the insulin level was 16.1 ± 1.4 ng/10^6^ cells (Fig.9A). The amount of C-peptide secreted was 0.24 ± 0.01 μg/10^6^ cells before induction. At a concentration of 5.5 mM, the C-peptide level was 0.89 ± 0.06 μg/10^6^ cells, at 15 mM it was 1.53 ± 0.11 μg/10^6^ cells and at 25 mM it was 1.30 ± 0.15 μg/10^6^ cells (Fig.9B). The intracellular insulin and C-peptide at each concentration of glucose versus cellular total protein are noted on figure 9C and D. Secreted insulin and C-peptide were proportional to glucose concentrations between 5.5-15 mM glucose whereas there was a decrease observed in insulin and C-peptide levels at the 25 mM glucose concentration.

## Discussion

Generation of insulin-producing cells from MSCs is new approach to treat type 1 diabetes (11, 12). Different cell sources such as cord blood, adipose tissue, and BM have been used to generate IPC (13-15). BM-MSCs generate more IPCs cells than adipose MSC, because bone marrow MSCs have more islet-like clusters and predominant pancreatic gene expression than adipose MSCs that differentiate into IPC (16).

Our result showed that, MSCs culture produced adherent spindle-like cells and expressed CD90 and CD105 MSC surface markers which confirmed the characterization of these cells. We examined the ability of MSCs to differentiate into adipogenic and osteogenic lineages. We evaluated the potential for BM-MSCs to generate morphologically and functionally pancreatic islet-like structures by genetic manipulation. Our findings revealed that *PDX1* made a strong change in gene MSCs and can induced its differentiation to a pancreatic lineage.

Lentiviral vectors are better than adenoviral vectors for gene delivery and therefore higher transgene expression levels have been observed. Lentiviral vectors can transfer the *PDX1* gene to MSCs in order to produce IPCs (17, 18). Karneili et al. performed differentiated human BM-MSCs in IPCs by over expression of rat Pdx1 gene and they showed expression of islet genes in manipulated BM-MSCs indicated a predisposition for differentiation toward phenotype of islet-cell (19). The homeodomain protein *PDX1* is a transcription factors possessing the hallmark 60 amino acid homeodomain that is highly conserved from yeast to human (20). Therefore, the rat Pdx1 gene can differentiate human MSC into IPCs. Transfection by lipofectamine is common in other studies, however we used calcium-phosphate method which generated a sufficient number of transduced cells. Cell-toxicity of the procedure according to the MTT assay showed that mortality at day 7 was not considerable, however significant mortality was observed at days 14 and 21. Our result showed that IPCs released insulin in response to physiological concentrations of glucose. Boroujeni and Aleyasin added B27, nicotinamide and beta fibroblast growth factor (βFGF) to the culture medium in order to promote insulin secretion (21). The current study did not use any chemicals or growth factors in the differentiation protocol. In this study IPCs secreted insulin in response to various concentrations of glucose (0, 5.5, 15, 25 mM) and peak insulin was 23 ng/l at the 15 mM glucose concentration. Peak insulin secretion in other studies was 6.85 ng/l (22), 40 ng/l (21) and 27 ng/l (19). C-peptide in the cell clusters confirmed that insulin release resulted from endogenous synthesis. Our findings showed that insulin was released from IPCs in response to increasing glucose levels up to 15 mM glucose, because the *PDX1* activated a number of genes involved in maintaining β-cell identity and function, such as insulin, Glut2 and GK, which suggested that these cells might have a glucose response (23), however, in current study poor response to high concentrations of glucose may be result from lack of expression gene encoding the K^+^ATP chanel subunits, KIR6.2 and SUR1 (19). In this study, the expression of the genes and protein related IPCs was detected by immunocytochemistry and RT-PCR. The *PDX1* gene expressed at the 7^th^ day of post induction whereas the *INSULIN* gene expressed at day 14 due to *INSULIN* gene activity-dependent *PDX1* gene activity (24). Morphological transformation initiated at day 5 and cells gradually accumulated together at day 14 with the formation of islet like clusters at day 21. The morphological changes in MSCs began from spindle-shaped to round shaped after day 5 post-induction. There were morphological changes to the IPCs at various days of differentiation, which was similar to pancreatic islet cells. The morphology changes was better in our study than other studies (19, 21).

## Conclusion

Our findings indicate that BM-MSCs can efficiently differentiate into IPCs efficiently by *PDX1* over-expression. Lentiviral vectors can deliver the *PDX1* gene to MSCs and induce pancreatic differentiation. We have used simple, efficient methods to generate effective IPCs. Additional experiments that include *in vivo* analyses are needed to confirm our findings.
